# Pancreatic Tuberculosis—A Condition That Mimics Pancreatic Cancer

**DOI:** 10.3390/medicina58091165

**Published:** 2022-08-27

**Authors:** Camelia Cristina Diaconu, Gina Gheorghe, Andreea Hortopan, Valentin Enache, Gabriela Ceobanu, Viorel Jinga, Cosmin Adrian, Vlad-Alexandru Ionescu

**Affiliations:** 1 Faculty of Medicine, University of Medicine and Pharmacy Carol Davila Bucharest, 050474 Bucharest, Romania; 2 Department of Internal Medicine, Clinical Emergency Hospital of Bucharest, 105402 Bucharest, Romania; 3 Academy of Romanian Scientists, 050045 Bucharest, Romania; 4 Department of Gastroenterology, Clinical Emergency Hospital of Bucharest, 105402 Bucharest, Romania; 5 Department of Anatomical Pathology, Clinical Emergency Hospital of Bucharest, 105402 Bucharest, Romania; 6 Department of Internal Medicine and Rheumatology, “Sfanta Maria” Clinical Hospital, 011172 Bucharest, Romania; 7 Department of Urology, “Professor Doctor Theodor Burghele” Hospital, 050653 Bucharest, Romania; 8 Department of Radiology, Clinical Emergency Hospital of Bucharest, 105402 Bucharest, Romania

**Keywords:** pancreatic tuberculosis, pancreatic cancer, epidemiology, diagnosis, prognosis

## Abstract

Tuberculosis is a disease with serious consequences in terms of morbidity and mortality. Pancreatic localization is very rare and is mostly encountered in patients with immunosuppressive disorders. A 59-year-old woman with arterial hypertension, grade 2 obesity, and a history of cholecystectomy, was admitted for fever (38.5 °C), jaundice, and marked physical asthenia. The blood tests showed severe metabolic acidosis, with partial respiratory compensation, mild microcytic normochromic anemia, inflammatory syndrome, procalcitonin value ten times the upper limit of normal, nitrogen retention syndrome, hypoalbuminemia, hypertriglyceridemia, hypercholesterolemia, and moderate hyponatremia. The electrocardiogram, chest X-ray, and abdominal ultrasound did not show any significant pathological changes. Contrast-enhanced computed tomography raised the suspicion of acute-on-chronic pancreatitis and subsequent evaluation by magnetic resonance imaging raised the suspicion of a pancreatic tumor. Pancreatic fine needle biopsy under echoendoscopic guidance revealed purulent material, which was sent for cytological and bacteriological examination. The Ziehl-Neelsen stain showed acid-alcoholic resistant bacilli, while bacterial cultures were positive for gentamicin and tigecycline-sensitive Klebsiella. The diagnosis of pancreatic tuberculosis was established. Pancreatic tuberculosis is a very rare condition that often mimics pancreatic cancer. The peculiarity of the case is the appearance of pancreatic tuberculosis in an immunocompetent woman and the association with Klebsiella infection.

## 1. Introduction

Tuberculosis is a condition caused by Mycobacterium tuberculosis, that most commonly affects the lungs [1]. All age groups are at risk of developing tuberculosis, but most cases of tuberculosis are reported among active adults. 98% of tuberculosis cases appear in low and middle-income countries [1]. Among the risk factors associated with this infection are conditions that lead to immunosuppression, malnutrition, alcohol consumption, and smoking [1]. Thus, patients with human immunodeficiency virus (HIV) infection have an 18 times higher risk of developing active tuberculosis, while alcohol consumption and smoking increase the risk of tuberculosis by 3.3 times and 1.6 times, respectively [1]. Currently, tuberculosis is the 13th leading cause of death and the second leading cause of death from infectious diseases worldwide, after COVID-19 [1]. In 2020, the World Health Organization (WHO) reported 1.5 million deaths from tuberculosis [1]. Of these, 214,000 deaths were among immunocompromised individuals, associated with HIV infection [1]. Regarding the incidence of tuberculosis, an annual decrease of approximately 2% was observed [1]. In the period 2015–2020, the cumulative reduction rate was 11% [1]. However, WHO reported 10 million new cases of tuberculosis worldwide in 2020 (5.6 million in men, 3.3 million in women, and 1.1 million in children) [1]. Of these, 1.9 million were attributed to malnutrition, 0.74 million to alcohol, and 0.73 million to smoking [1].

In Romania, the overall incidence of tuberculosis decreased by 27.9% between 1995 and 2016 (102.6 cases per 100,000 inhabitants in 1995 vs. 74 cases per 100,000 inhabitants in 2016) [2]. In 2020, WHO reported an incidence of 64 cases per 100,000 Romanian inhabitants [3].

In most cases of tuberculosis, the infection is localized in the lungs. Only 12.5% of cases have extrapulmonary involvement, and of these 11–16% have abdominal localization [4,5]. Possible abdominal sites include the stomach, intestinal tract, pancreas, liver, bile duct, spleen, and lymph nodes [6]. Regarding the routes of transmission of Mycobacterium tuberculosis to these abdominal sites, the literature reports hematogenous transmission from the primary lung site, direct invasion from an adjacent organ, ingestion of infected sputum among patients with pulmonary tuberculosis, or ingestion of infected milk products [6].

Pancreatic tuberculosis is one of the rarest forms of tuberculosis, and it presents with non-specific clinical manifestations. However, recently there has been an increase in the number of cases of tuberculosis with pancreatic localization [7,8,9,10]. This may be due to the development of new diagnostic imaging techniques, the increasing availability of methods that allow obtaining pancreatic specimens, the increase in the number of immunocompromised patients, as well as an increase in HIV infections and advanced stage malignancies [11]. Due to the low incidence and non-specific symptoms, the approach to the diagnosis of pancreatic tuberculosis is difficult. This infectious disease often mimics pancreatic cancer [12]. Pharmacological treatment for pancreatic tuberculosis has been reported to be very effective in most cases [12]. Under these conditions, rapid diagnostic confirmation of pancreatic tuberculosis leads to a reduction in the costs of caring for these patients, allowing the avoidance of surgical procedures [12].

## 2. Case Presentation

**Figure 1:**
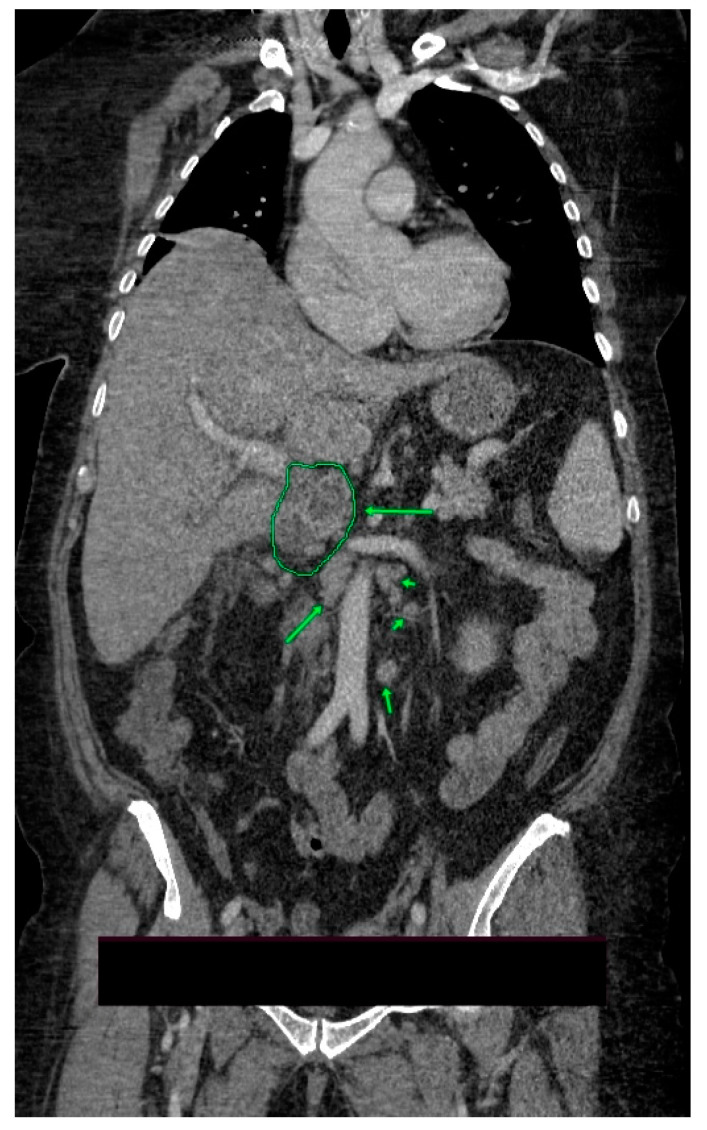
Contrast-enhanced abdominal pelvic computed tomography, venous phase, coronal imaging plane: globally enlarged, irregularly shaped, inhomogeneous cephalic contours of the pancreas, multiple periaortic and interaortocaval lymph nodes.

A 59-year-old female, known with stage 2 arterial hypertension, grade 2 obesity, with a history of cholecystectomy, was admitted for fever (38.5 °C), jaundice, and marked physical asthenia. The symptoms began about 3 weeks before admission and gradually worsened. The patient denied any significant family medical history. Upon examination, the patient was ill-appearing, conscious, with icteric skin, sclera, and mucous membranes, without any palpable lymph nodes; lung sounds were clear bilaterally, oxygen saturation 98–100% with additional oxygen on face mask; blood pressure of 98/58 mmHg and heart rate of 102 beats per minute; non-tender abdomen; acholic stool and hyperchromic urine. The neurological examination revealed no focal deficit. The laboratory tests showed severe metabolic acidosis with partial respiratory compensation, mild microcytic normochromic anemia, elevated acute phase reactants, procalcitonin ten times the upper normal limit, azotemia, hypoalbuminemia, hypertriglyceridemia, hypercholesterolemia, and moderate hyponatremia. The electrocardiogram and chest X-ray revealed normal findings. Abdominal ultrasound identified a normal-looking liver, without dilatations of intrahepatic or extrahepatic bile ducts, a spleen of 13 cm, without other pathological changes. Based on these results, the diagnosis of sepsis of unknown origin, jaundice, and acute renal failure was established. A gastroenterology consultation was requested, that excluded liver cirrhosis (due to the lack of clinical, serological, and imaging criteria), obstructive jaundice, and angiocolitis (absence of biliary ducts dilatations and biliary stones). The association of cholestasis, hepatocytolysis, and azotemia raised the suspicion of leptospirosis. This diagnosis was later refuted by performing anti-leptospira antibodies, with a negative result. A few hours after admission, the patient’s condition deteriorated, manifesting signs of septic distributive shock. The patient was transferred to the intensive care unit, where her condition remained severe, with hemodynamic instability and need for vasopressor support with norepinephrine in moderate doses. Infectious screening was performed and broad-spectrum antibiotherapy was initiated. Moreover, the CA 19-9 level was dosed, with values 20 times the upper normal limit, and CA-125 with values five times the upper normal limit. Contrast-enhanced abdominal pelvic computed tomography revealed a globally enlarged, irregularly shaped, inhomogeneous cephalic contours of the pancreas, with hypocaptive images and microcalcifications; peripancreatic fluid collections with a diameter of up to 2.5 cm, two cystic images with a diameter of 2.4 cm and 2.7 cm, respectively, that come in contact with the portal vein and the head of the pancreas (small pseudocysts), as well as multiple periaortic and interaortocaval lymph nodes with a maximum axial diameter of 17 mm (Figure 1). These findings raised the suspicion of acute-on-chronic pancreatitis.

**Figure 2:**
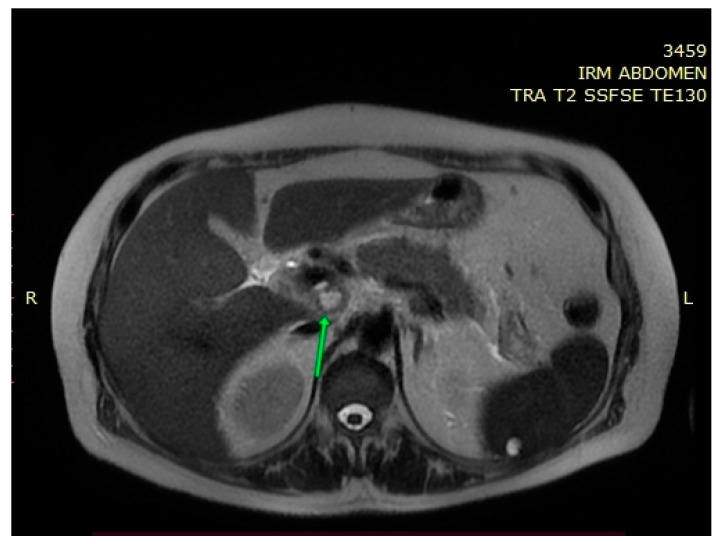
MRI—T2 weighted image: a multicystic cephalopancreatic lesion.

**Figure 3:**
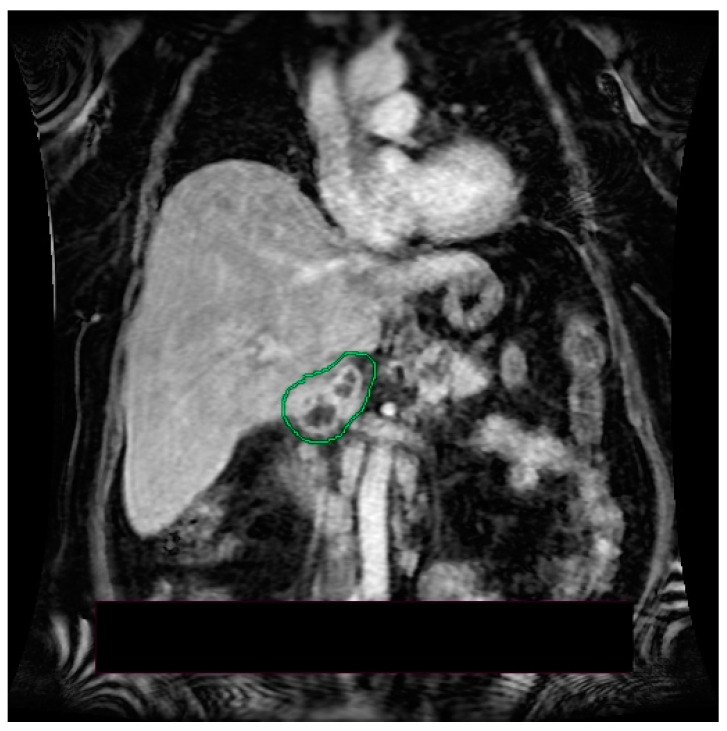
MRI—T1 post contrast sequence, coronal imaging plane: inhomogeneous cephalopancreatic lesion.

**Figure 4:**
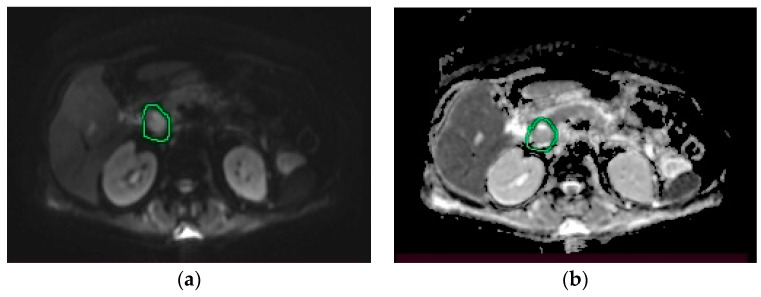
MRI. (**a**) Diffusion-weighted imaging (DWI) sequence: hyperintense cephalopancreatic lesion; (**b**) apparent diffusion coefficient (ADC) sequence: hypointense cephalopancreatic lesion.

Under broad-spectrum antibiotic, antifungal, and vasopressor treatment, fluid and electrolyte balance restoration treatment, analgesic, antipyretic and oxygen therapy, the patient’s general condition improved, allowing the transfer to the internal medicine ward. The patient was hemodynamically stable, with normochromic urine and normal-looking stool. Laboratory investigations revealed mildly elevated aminotranspherases, moderate to severe anemia (without bleeding signs), normal white blood cell count, and no cholestasis. The upper endoscopy identified class A Los Angeles esophagitis; the colonoscopy had normal findings. During hospitalization, the patient continued to have multiple episodes of fever; blood cultures and urine cultures were collected. Blood cultures were positive for Acinetobacter, and antibiotic treatment according to antibiogram was initiated, with persistent fever. Infectious screening was continued, showing negative results for urine cultures, samples were collected from the top of the central venous catheter, and blood cultures. Echocardiography was also performed and ruled out endocarditis. To further evaluate the pancreatic lesions, nuclear magnetic resonance imaging (MRI) was performed, revealing a multicystic pancreatic lesion that raised the suspicion of a pancreatic tumor (Figure 2, Figure 3 and Figure 4).

**Figure 5:**
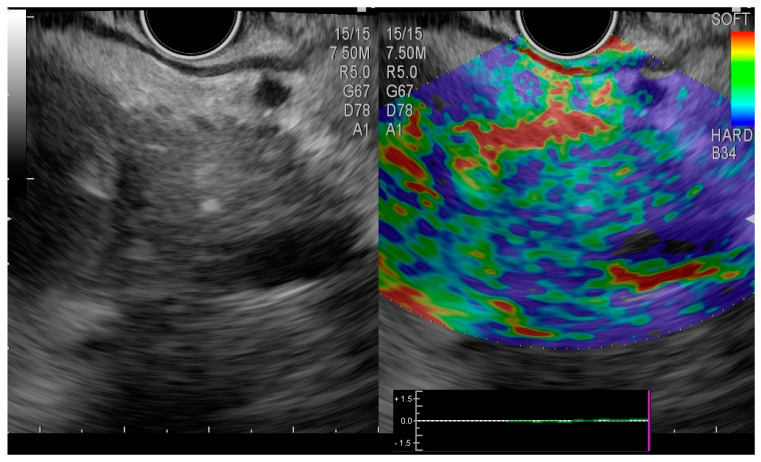
Endoscopic ultrasound elastography. Hypoechoic cephalic pancreatic lesion with mixed pattern.

The echoendoscopy revealed changes suggestive of chronic pancreatitis, but also a cephalic pancreatic lesion whose differential diagnosis included cystadenoma, cystadenocarcinoma, and infected pseudocyst (Figure 5).

**Figure 6:**
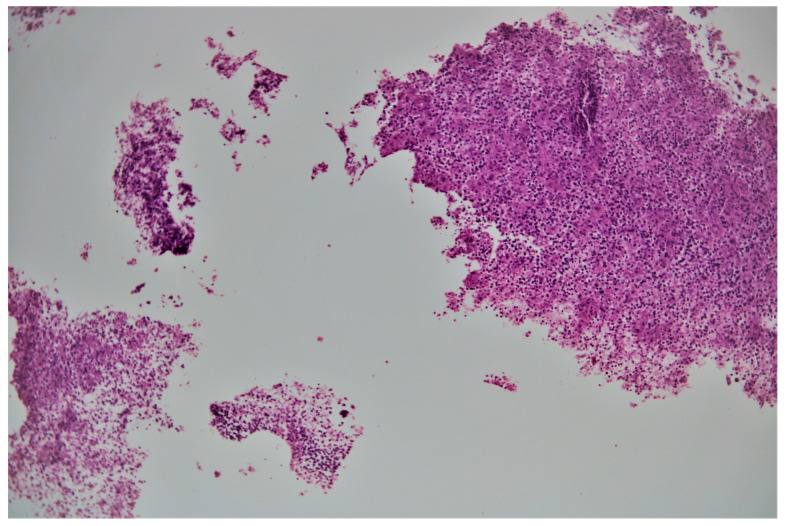
100× biopsy fragments with necrotic material and inflammatory cells.

**Figure 7:**
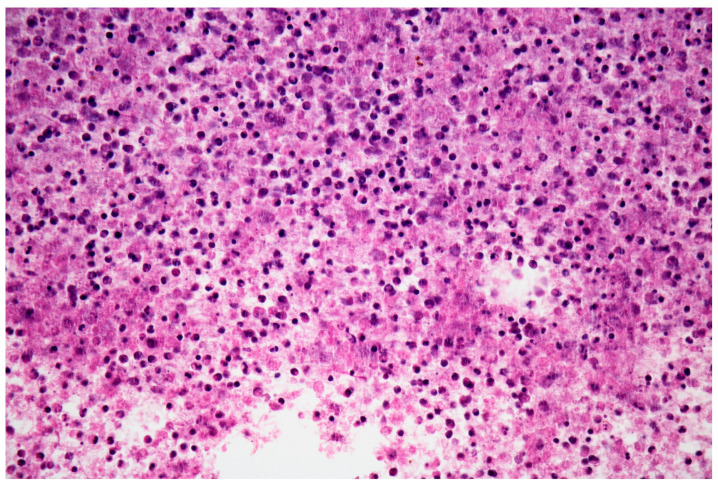
Hematoxylin-eosin stain 400×: necrotic material with lymphocytes, polymorphonuclear cells and cell debris.

A fine needle pancreatic biopsy was performed under echoendoscopic guidance, with extraction of purulent material, that was sent for cytological and bacteriological examination. The Ziehl-Neelsen stain highlighted acid-alcoholic resistant bacilli (300 AARB/300 fields), while bacterial cultures were positive for gentamicin and tigecycline-sensitive Klebsiella. The histological examination identified necrotic and cellular detritus, polymorphonuclear cells, neutrophils, and lymphocytes, without epithelial cells (Figure 6 and Figure 7). At this moment, the patient was transferred to a tuberculosis clinic, where tuberculostatic treatment was initiated. The clinical evolution of the patient under tuberculostatic treatment was favorable.

## 3. Discussion

Tuberculosis has remained a major cause of morbidity and mortality in developing countries. Pancreatic tuberculosis is very rare and, in most cases, has been reported in immunosuppressed patients or in the context of disseminated disease [13]. The low probability of Mycobacterium tuberculosis localization at the pancreatic level could be explained by the retroperitoneal location of the pancreas and the antimicrobial properties of its enzymes, such as lipase and deoxyribonucleases extracts [14,15]. The diagnosis of pancreatic tuberculosis may be hampered by the difficulty of detecting acid-fast bacilli (AFB) in the biopsy sample, as most forms of abdominal tuberculosis are paucibacillary [16]. Under these conditions, the particularities of our case consist, on the one hand, in the appearance of pancreatic tuberculosis in an immunocompetent patient, and on the other hand, in the detection of AFB in the intracystic fluid. Sohni et al. reported a similar case, of pancreatic tuberculosis in an immunocompetent patient by detecting AFB in tissue samples obtained by fine-needle aspiration (FNA) and growth in culture of Mycobacterium tuberculosis [13]. Abbaszadeh et al. presented another case of pancreatic tuberculosis in a 23-year-old woman without significant comorbidities. In this case, the diagnosis was established after obtaining the tissue sample by exploratory laparotomy [15].

Regarding the age and sex of patients with pancreatic tuberculosis, according to data from the literature, most cases occur in men in the fourth to fifth decade of life [17,18,19]. This could be explained by the higher prevalence of risk factors, such as smoking or alcohol consumption, in this category of patients. Contrary to the literature data, in our case, pancreatic tuberculosis developed in an immunocompetent woman, without a history of smoking or alcohol consumption.

Clinical manifestations are usually nonspecific, which may delay the diagnosis [20,21]. Patients with pancreatic tuberculosis may have abdominal pain, fever and night sweats, loss of appetite, weight loss, jaundice, diarrhea, or peripheral lymphadenopathy. These symptoms could be explained both by the systemic effects of tuberculosis and by the local changes in the pancreas and its adjacent structures [22]. Abdominal pain has no particular characteristics and its presence varies between 28.6% and 100% [23]. A meta-analysis of 116 studies and 166 patients with pancreatic tuberculosis reported abdominal pain in 74.8% of cases, fever in 46.5% of cases, weight loss in 51.6% of cases, jaundice in 20% of cases, and diarrhea in 3.1% of cases [6]. Jaundice may be secondary to biliary obstruction by pancreatic mass or adjacent lymphadenopathy [12,23].

Other clinical presentations in patients with pancreatic tuberculosis, rarely reported, are dyspepsia [24], signs of acute or chronic pancreatitis [25], secondary diabetes mellitus [26], and gastrointestinal bleeding secondary to splenic vein thrombosis [27]. In our case, the patient presented two of the symptoms reported as common in patients with pancreatic tuberculosis, namely fever and jaundice. The absence of the epidemiological context and risk factors for tuberculosis, however, led to a small delay in establishing a positive diagnosis.

Laboratory tests may show anemia, changes in biological markers that assess liver function, changes in serum bilirubin or glucose levels [28,29]. Moreover, data from the literature suggest a low diagnostic value for serum amylase or lipase levels, which are increased only in a limited number of patients with pancreatic tuberculosis (26.8% and 31.3%, respectively) [6]. Data on impaired exocrine and endocrine pancreatic function in patients with pancreatic tuberculosis are limited. Jeon and Murray have shown an increased risk of active tuberculosis in patients with diabetes mellitus [30]. One hypothesis that could explain this association is the diabetes-induced disruption of the immune response needed to prevent bacterial proliferation [31,32]. Some data support the possibility of diabetes onset or worsening due to lesions in the pancreatic parenchyma, secondary to pancreatic tuberculosis [33].

The imaging methods that can be used to diagnose pancreatic tuberculosis are abdominal ultrasonography, CT, MRI, endoscopic retrograde cholangiopancreatography (ERCP), and endoscopic ultrasound (EUS) [6]. The radiological changes can be classified into three categories: mass-forming, nodular form, or diffuse form [34]. In most cases, imaging in pancreatic tuberculosis reveals the presence of a pancreatic mass (79.5%) located most frequently in the pancreatic head (59%) [6]. More rarely, this pseudotumor can be located in the pancreatic body (18.2%), tail (13.4%), or neck (1.8%) [6]. CT scan was performed in almost all patients with pancreatic tuberculosis reported in the literature [35]. In most cases, it did not establish the diagnosis because there were no pathognomonic radiological features for pancreatic tuberculosis [35]. Nagar et al. conducted a study on 32 patients with pancreatic tuberculosis. CT examination revealed pancreatic hypodense collections associated with pancreatic lymphadenopathy in 29 patients [29]. The remaining three patients presented with pancreatic mass lesions [29]. Unlike adenocarcinoma, in pancreatic tuberculosis the pancreatic duct and common bile duct usually show no changes [36]. However, in the diffuse form of pancreatic tuberculosis, changes in the size of the main pancreatic duct, bile ducts, and local vascular invasion may occur [36]. Under these conditions, changes in the pancreatic duct or main bile duct do not rule out the presence of pancreatic tuberculosis [36]. Another radiological finding that can mimic pancreatic cancer is the presence of calcifications inside the pancreatic lesions [35,37]. These were found to be present in 7.1–56.3% of cases [35,37]. In our case, no changes in the size of the Wirsung duct were found, but calcifications were identified.

MRI can also provide valuable information to help establish the diagnosis of pancreatic tuberculosis [12]. De Backer et al. concluded that in cases of focal pancreatic tuberculosis, MRI evaluation may reveal a delineated pancreatic mass, with heterogeneous enhancement, most commonly located in the head of the pancreas [38]. In diffuse forms of pancreatic tuberculosis, MRI evaluation may show an overall increase in pancreatic size, with narrowing of the Wirsung duct and heterogeneous enhancement [38]. In T1 sequences, the lesions usually appear hypointense and in T2 sequences, hyperintense [17,39]. Peripancreatic lymphadenopathy can be frequently identified [12]. In our case, MRI identified a multicystic mass in medial contact with the pancreatic cephalic region. Considering its behavior in diffusion and postcontrast sequences, it raises the suspicion of pancreatic tumor. Peripancreatic lymphadenopathy, most commonly grouped in the vicinity of the left renal artery, was also identified.

ERCP is not commonly used in the management of patients with pancreatic tuberculosis. In case of the development of complications such as pancreaticobiliary fistulas or biliary obstruction, this imaging investigation can be useful both for diagnostic and therapeutic purposes [39,40]. Bioptic samples can be obtained by ERCP-guided brushing of the bile ducts from which AFB can be isolated.

Currently, the imaging method of choice in patients with suspected pancreatic tuberculosis is EUS [41]. In addition to the imaging evaluation of the pancreas and peripancreatic lymph nodes, it allows the collection of biopsy samples by fine-needle aspiration/biopsy (FNA/FNB-EUS) [41]. Rana et al. compared the images obtained by echoendoscopy in two groups of patients: one group of 6 patients with pancreatic tuberculosis and the other group of 25 patients with pancreatic adenocarcinoma [42]. The differences were not significant. However, the age of patients with pancreatic cancer tended to be higher compared to those with pancreatic tuberculosis. Pancreatic cancer patients also had higher serum bilirubin levels, as well as more frequent changes in the size of the main duct bile and Wirsung duct [42]. FNA is the most feasible, non-invasive method that allows the collection of a tissue sample and subsequent histopathological examination [43,44]. The risk of sampled cells or microorganisms spreading is minor [44]. Bioptic samples from pancreatic lesions can also be obtained by CT-guided biopsy or laparoscopy [6]. In our case, FNA-EUS allowed the diagnosis of pancreatic tuberculosis.

Clinical manifestations in association with imaging findings in patients with pancreatic tuberculosis frequently lead to diagnostic confusion, as pancreatic tuberculosis may mimic pancreatic cancer. The only way of establishing a definite diagnosis is by collecting biopsy samples with subsequent histopathological examination, Ziehl-Neelsen staining, cultures, and polymerase chain reaction (PCR). These diagnostic methods may reveal the presence of Mycobacterium tuberculosis; the histopathological examination usually reveals necrotizing granuloma [45]. The diagnostic value of PCR was found to be higher (46–96%) than the diagnostic value of AFB smear (0–62%) or cultures (19–81%) [46]. In our case, the histopathological examination identified only nonspecific inflammatory changes, but the Ziehl-Neelsen staining identified AFB.

Regarding extrapancreatic involvement, most patients with tuberculosis have peripancreatic lymphadenopathy [47]. These changes are also common in patients with pancreatic cancer, making the diagnostic process more difficult [47,48]. Imaging studies in our patient revealed a hypoechoic mass in the head of the pancreas and peripancreatic lymphadenopathy, which initially led to the suspicion of pancreatic cancer. A meta-analysis of 166 patients with pancreatic tuberculosis also reported extrapancreatic involvement of the spleen (8.3%), intestinal tract (8.2%), liver (6.9%), and lungs (6.3%) [6].

## 4. Conclusions

In conclusion, the particularities of our case consist in the presence of pancreatic tuberculosis in an immunocompetent patient, with an initial severe presentation, and also the association of pancreatic infection with Klebsiella. Establishing a positive diagnosis proved difficult, as the patient initially showed a diffuse form of pancreatic tuberculosis, with subsequent localization. The differential diagnosis included acute-on-chronic pancreatitis and pancreatic cancer. However, the patient showed no risk factors for pancreatitis or other signs of neoplastic disease. Although the histopathological diagnosis identified only nonspecific inflammatory changes, the Ziehl-Neelsen stain highlighted AFB. Like other cases of pancreatic tuberculosis reported in the literature, the diagnostic process was challenging, despite the accessibility of various diagnostic tools.

## Data Availability

Not applicable.

## References

[1] World Health Organization. https://www.who.int/news-room/fact-sheets/detail/tuberculosis.

[2] Golli A.L., Nitu M.F., Turcu F., Popescu M., Ciobanu-Mitrache L., Olteanu M. (2019). Tuberculosis remains a public health problem in Romania. Int. J. Tuberc. Lung Dis..

[3] Incidence of Tuberculosis (per 100,000 People)—Romania. https://data.worldbank.org/indicator/SH.TBS.INCD?locations=RO.

[4] Singhal A., Gulati A., Frizell R., Manning A.P. (2005). Abdominal tuberculosis in Bradford, UK: 1992–2002. Eur. J. Gastroenterol. Hepatol..

[5] Misra S.P., Misra V., Dwivedi M., Gupta S.C. (1999). Colonic tuberculosis: Clinical features, endoscopic appearance and management. J. Gastroenterol. Hepatol..

[6] Panic N., Maetzel H., Bulajic M., Radovanovic M., Lohr J.M. (2020). Pancreatic tuberculosis: A systematic review of symptoms, diagnosis and treatment. United Eur. Gastroenterol. J..

[7] Sun P.J., Lin Y., Cui X.J. (2018). Isolated pancreatic tuberculosis with elevated CA 19-9 levels masquerading as a malignancy: A rare case report and literature review. Medicine.

[8] Singhai P., Gadhadh R., Joshi S., Krishnan S., Aparna C. (2017). Isolated pancreatic tuberculosis in an immunocompetent host. J. Assoc. Physicians India.

[9] Falkowski A.L., Graber J., Haack H.G., Tarr P.E., Rasch H. (2013). Isolated pancreatic tuberculosis: A case report and radiological comparison with cystic pancreatic lesions. J. Radiol. Case Rep..

[10] Rana S., Sharma V., Sharma R., Bhasin D. (2016). Involvement of mediastinal/intra-abdominal lymph nodes, spleen, liver, and left adrenal in presumed isolated pancreatic tuberculosis: An endoscopic ultrasound study. J. Dig. Endosc..

[11] Hesseling A.C., Rabie H. (2016). Tuberculosis and HIV remain major causes of death in African children. Int. J. Tuberc. Lung Dis..

[12] Sharma V., Rana S.S., Kumar A., Bhasin D.K. (2016). Pancreatic tuberculosis. J. Gastroenterol. Hepatol..

[13] Sohni D. (2021). Pancreatic mass: Include yuberculosis in the differential diagnosis. Cureus.

[14] Cho S.B. (2009). Pancreatic tuberculosis presenting with pancreatic cystic tumor: A case report and review of the literature. Korean J. Gastroenterol..

[15] Abbaszadeh M., Rezai J., Hasibi M., Larry M., Ostovaneh M.R., Javidanbardan S., Mirbagheri S.A. (2017). Pancreatic tuberculosis in an immunocompetent patient: A case report and review of the literature. Middle East J. Dig. Dis..

[16] Veerabadran P., Sasnur P., Subramanian S., Marappagounder S. (2007). Pancreatic tuberculosis-abdominal tuberculosis presenting as pancreatic abscesses and colonic perforation. World J. Gastroenterol..

[17] Kim J.B., Lee S.S., Kim S.H., Byun J.H., Park D.H., Lee T.Y., Lee B.U., Jeong S.U., Seo D.W., Lee S.K. (2014). Peripancreatic tuberculous lymphadenopathy masquerading as pancreatic malignancy: A single-center experience. J. Gastroenterol. Hepatol..

[18] Xia F., Poon R.T., Wang S.G., Bie P., Huang X.Q., Dong J.H. (2003). Tuberculosis of pancreas and peripancreatic lymph nodes in immunocompetent patients: Experience from China. World J. Gastroenterol..

[19] Ibrahim G.F., Al-Nakshabandi N.A. (2011). Pancreatic tuberculosis: Role of multidetector computed tomography. Can. Assoc. Radiol. J..

[20] Rana S.S., Sharma V., Sampath S., Sharma R., Mittal B.R., Bhasin D.K. (2014). Vascular invasion does not discriminate between pancreatic tuberculosis and pancreatic malignancy: A case series. Ann. Gastroenterol..

[21] Margekar S.L., Meena R.K., Kapoor S., Dhamija R.K. (2021). Pancreatic tuberculosis: An unusual presentation. Natl. Med. J. India.

[22] Fogel E.L., Shahda S., Sandrasegaran K., DeWitt J., Easler J.J., Agarwal D.M., Eagleson M., Zyromski N.J., House M.G., Ellsworth S. (2017). A multidisciplinary approach to pancreas cancer in 2016: A review. Am. J. Gastroenterol..

[23] Yan C.Q., Guo J.C., Zhao Y.P. (2007). Diagnosis and management of isolated pancreatic tuberculosis: Experience of 13 cases. Chin. Med. Sci. J..

[24] Pandita K.K., Sarla, Dogra S. (2009). Isolated pancreatic tuberculosis. Indian J. Med. Microbiol..

[25] Rushing J.L., Hanna C.J., Selecky P.A. (1978). Pancreatitis as the presenting manifestation of military tuberculosis. West. J. Med..

[26] Patankar T., Prasad S., Laxminarayan R. (1999). Diabetes mellitus: An uncommon manifestation of tuberculosis. J. Assoc. Physicians India.

[27] Fan S.T., Yan K.W., Lau W.Y., Wong K.K. (1986). Tuberculosis of the pancreas: A rare cause of massive gastrointestinal bleeding. Br. J. Surg..

[28] Auerbach O. (1944). Acute generalized Miliary Tuberculosis. Am. J. Pathol..

[29] Nagar A.M., Raut A.A., Morani A.C., Sanghvi D.A., Desai C.S., Thapar V.B. (2009). Pancreatic tuberculosis: A clinical and imaging review of 32 cases. J. Comput. Assist. Tomogr..

[30] Jeon C.Y., Murray M.B. (2008). Diabetes mellitus increases the risk of active tuberculosis: A systematic review of 13 observational studies. PLoS Med..

[31] Martens G.W., Arikan M.C., Lee J., Ren F., Greiner D., Kornfeld H. (2007). Tuberculosis susceptibility of diabetic mice. Am. J. Respir. Cell Mol. Biol..

[32] Viardot A., Grey S.T., Mackay F., Chisholm D. (2007). Potential antiinflammatory role of insulin via the preferential polarization of effector T cells toward a T helper 2 phenotype. Endocrinology.

[33] Pande T., Huddart S., Xavier W., Kulavalli S., Chen T., Pai M., Saravu K. (2018). Prevalence of diabetes mellitus amongst hospitalized tuberculosis patients at an Indian tertiary care center: A descriptive analysis. PLoS ONE.

[34] Jemni I., Akkari I., Mrabet S., Jazia E.B. (2020). Isolated pancreatic tuberculosis mimicking pancreatic cancer in an immunocompetent host: An elusive diagnosis. Radiol. Case Rep..

[35] Chaudhary P., Bhadana U., Arora M.P. (2015). Pancreatic tuberculosis. Indian J. Surg..

[36] Shahrokh S., Miri M.B., Safari M.T., Alizadeh A.H.M. (2015). Pancreatic tuberculosis: An overview. JOP J. Pancreas.

[37] Franco-Paredes C., Leonard M., Jurado R., Blumberg H.H., Smith R.M. (2002). Tuberculosis of the pancreas: Report of two cases and review of the literature. Am. J. Med. Sci..

[38] De Backer A.I., Mortele K.J., Bomans P., De Keulenaer B.L., Vanschoubroeck I.J., Kockc M.M. (2005). Tuberculosis of the pancreas: MRI features. Am. J. Roentgenol..

[39] Nakai Y., Tsujino T., Kawabe T., Kogure H., Sasaki T., Yamamoto N., Sasahira N., Isayama H., Tada M., Omata M. (2007). Pancreatic tuberculosis with a pancreaticobiliary fistula. Dig. Dis. Sci..

[40] Sachdev A., D’Cruz S., Chauhan S., Thakur R., Kapoor V., Handa U. (2006). Pancreaticobiliary tuberculosis diagnosed by endoscopic brushings. J. Pancreas.

[41] Hoilat G.J., Abdu M., Hoilat J., Gitto L., Bhutta A.Q. (2020). A Rare Case of Pancreatic Tuberculosis Diagnosed via Endoscopic Ultrasound-Guided Fine Needle Aspiration and Polymerase Chain Reaction. Cureus.

[42] Rana S.S., Bhasin D.K., Srinivasan R., Sampath S., Mittal B.R., Singh K. (2012). Distinctive endoscopic ultrasound features of isolated pancreatic tuberculosis and requirements for biliary stenting. Clin. Gastroenterol. Hepatol..

[43] Chatterjee S., Schmid M.L., Anderson K., Oppong K.W. (2012). Tuberculosis, and the pancreas: A diagnostic challenge solved by endoscopic ultrasound. A case series. J. Gastrointest Liver Dis..

[44] Song T.J., Lee S.S., Park D.H., Lee T.Y., Lee S.O., Seo D.W., Lee S.K., Kim M.H. (2009). Yield of EUS-guided FNA on the diagnosis of pancreatic/peripancreatic tuberculosis. Gastrointest Endosc..

[45] Chaudhary A., Negi S.S., Sachdev A.K., Gondal R. (2002). Pancreatic tuberculosis: Still a histopathological diagnosis. Dig. Surg..

[46] Arai J., Kitamura K., Yamamiya A., Ishii Y., Nomoto T., Honma T., Ishida H., Shiozawa E., Takimoto M., Yoshida H. (2017). Peripancreatic tuberculous lymphadenitis diagnosed via endoscopic ultrasound-guided fine-needle aspiration and polymerase chain reaction. Intern. Med..

[47] Chu L.C., Goggins M.G., Fishman E.K. (2017). Diagnosis and detection of pancreatic cancer. Cancer J..

[48] Gheorghe G., Bungau S., Ilie M., Behl T., Vesa C.M., Brisc C., Bacalbasa N., Turi V., Costache R.S., Diaconu C.C. (2020). The early diagnosis of pancreatic cancer: The key for survival. Diagnostics.

